# Non-Uniform Dispersion of the Source-Sink Relationship Alters Wavefront Curvature

**DOI:** 10.1371/journal.pone.0078328

**Published:** 2013-11-04

**Authors:** Lucia Romero, Beatriz Trenor, Jose M. Ferrero, C. Frank Starmer

**Affiliations:** 1 Instituto de Investigación Interuniversitario en Bioingeniería y Tecnología Orientada al Ser Humano (I3BH), Universitat Politècnica de València, Valencia, Valencia, Spain; 2 Cardiovascular and Metabolic Disorders, Duke-NUS Graduate Medical School, Singapore; 3 Biostatistics and Bioinformatics, Duke University, Durham, North Carolina, United States of America; Georgia State University, United States of America

## Abstract

The distribution of cellular source-sink relationships plays an important role in cardiac propagation. It can lead to conduction slowing and block as well as wave fractionation. It is of great interest to unravel the mechanisms underlying evolution in wavefront geometry. Our goal is to investigate the role of the source-sink relationship on wavefront geometry using computer simulations. We analyzed the role of variability in the microscopic source-sink relationship in driving changes in wavefront geometry. The electrophysiological activity of a homogeneous isotropic tissue was simulated using the ten Tusscher and Panfilov 2006 action potential model and the source-sink relationship was characterized using an improved version of the Romero et al. safety factor formulation (SF_m2_). Our simulations reveal that non-uniform dispersion of the cellular source-sink relationship (dispersion along the wavefront) leads to alterations in curvature. To better understand the role of the source-sink relationship in the process of wave formation, the electrophysiological activity at the initiation of excitation waves in a 1D strand was examined and the source-sink relationship was characterized using the two recently updated safety factor formulations: the SF_m2_ and the Boyle-Vigmond (SF_VB_) definitions. The electrophysiological activity at the initiation of excitation waves was intimately related to the SF_m2_ profiles, while the SF_VB_ led to several counterintuitive observations. Importantly, with the SF_m2_ characterization, a critical source-sink relationship for initiation of excitation waves was identified, which was independent of the size of the electrode of excitation, membrane excitability, or tissue conductivity. In conclusion, our work suggests that non-uniform dispersion of the source-sink relationship alters wavefront curvature and a critical source-sink relationship profile separates wave expansion from collapse. Our study reinforces the idea that the safety factor represents a powerful tool to study the mechanisms of cardiac propagation in silico, providing a better understanding of cardiac arrhythmias and their therapy.

## Introduction

In the heart, pronounced curvatures of excitation wavefronts can be observed when waves are initiated by small electrodes, in the case of waves emerging from narrow tissue structures, or in waves propagating around sharp edges of anatomical obstacles or around a zone of functional conduction block during spiral wave rotation [1], [2]. It has been demonstrated that wavefront curvature is associated with non-uniform propagation of a cardiac impulse [1]–[5] potentially leading to the development of lethal cardiac arrhythmias, such as ventricular tachycardia or ventricular fibrillation [3], [6]. Indeed, high curvature has been associated with conduction block and with the dynamic behavior of spiral waves [2]. Convex wavefronts cause slowing of propagation because the local excitatory current supplied by the cells at the front of the waves (electrical source) diverges into a larger area downstream (electrical sink) [1]–[3].

It is well known that the source-sink relationship determines movement of a wavefront. Reduction of the availability of source charge or increase of the sink charge required to excite the cell may produce propagation failure. Recently, a parameter that characterizes the source-sink relationship called the safety factor of propagation (SF) has been mathematically formulated. Although the first quantitative formulation of the SF for one-dimensional fibers was proposed in 1990 by Delgado et al. [7], it was not until 1997 that Shaw and Rudy proposed a formulation for the SF that decreased its magnitude with membrane excitability reduction and that dropped below unity with conduction block [8]. Then, this SF definition was modified to enable its use in inhomogeneous cardiac one-dimensional tissues [9]. In 2005, our group optimized the formulation by Shaw and Rudy in order to save computational resources and to allow its use in two-dimensional tissues [10], [11]. More recently, Boyle and Vigmond proposed an intuitive SF formulation suitable for any dimension [12].

The SF has been used to theoretically investigate the ionic mechanisms of cardiac propagation and arrhythmias under different conditions, such as slow conduction and conduction path branching in the heart [8], [13], reentry generation [11], wavefront-obstacle interactions [14], conduction through the Purkinje-ventricular junction (PVJ) [12], [15], [16] and in cardiac tissues with microstructural variations [17]. Here, to gain insights into the mechanisms of wavefront conformation we improve our previous SF formulation and we use it to characterize the source-sink relationship of excitatory waves in a 2D tissue. We hypothesized that the non-uniform distribution of source-sink relationship along a propagating wave would lead to transition from a uniform wavefront to a non-uniform wavefront. To explore this hypothesis, we analyzed the source-sink relationship in cardiac tissue during wavefront evolution. To better understand the role of the source-sink relationship in the process of wave formation, the SF_m2_ was computed at the initiation of excitation in a 1D strand. Our results show that non-uniform dispersion of the source-sink relationship as characterized by SF_m2_ alters wavefront curvature. Moreover, propagation succeeds when the source-sink characterization exceeds a critical SF_m2_ spatial profile, regardless of the stimulation electrode size, the membrane excitability and the tissue conductivity.

## Materials and Methods

### Tissue model

Membrane kinetics were simulated using the ten Tusscher and Panfilov 2006 human ventricular action potential (AP) model (TP06 model) [18].

The reaction–diffusion equation (Eq. 1) governs the electrical propagation in the simulated monodomain tissue,
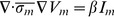
(1)where V_m_ is the transmembrane potential, 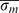 is the conductivity tensor, β is the surface-to-volume ratio and I_m_ is the membrane current. The nominal tissue conductivity was set to 0.6 S/m, which yields a conduction velocity of 50 cm/s. A 10 mm×10 mm isotropic tissue and a 20 mm strand were simulated. Element edge lengths were 20 µm and 100 µm and the time step was fixed to 0.01 ms and 0.002 ms, respectively. The operator splitting technique was applied to the monodomain equations. “No-flux” boundary conditions were applied. To reduce membrane excitability, the maximum sodium conductance (g_Na_) was multiplied by 0.5, and to reduce the intercellular coupling, the tissue conductivity was multiplied by 0.25. In addition to the aforementioned simulation conditions, the 1D tissue was also simulated under more severe conditions. Indeed, g_Na_ was multiplied by 0.3 or the tissue conductivity was multiplied by 0.125. Higher spatial and temporal discretization was needed for numeric accuracy to simulate reduced tissue conductivity in the 1D strand. Indeed, 75% and 87.5% tissue conductivity reductions were simulated using element edge lengths and time step durations five and ten times smaller than in control conditions, respectively.

### Stimulation protocol

The 2D tissue was stimulated using four different electrodes: a 10 mm×0.1 mm, and a 3 mm×0.1 mm electrode as well as a 0.5 mm and a 1.5 mm radius circular shaped electrode located in the center of the tissue.

The 1D tissue was also stimulated using electrodes of different sizes (4, 8 and 12 mm) in the middle of the strand. Stimulation consisted on a rectangular pulse of 2 ms in duration and a large number of simulations were run in the 1D tissue for every simulation condition to find the threshold amplitude current with a precision of 10^−15^ µA/µF. Stimulation current was applied after 30 ms of electrical rest to allow membrane stabilization.

### Definition of basic parameters

The activation time (AT) was defined as the instant of maximal depolarization velocity of the membrane potential.

The source-sink relationship was analyzed using the two most updated SF formulations: an improved version (SF_m2_) of the Romero and coworkers (SF_m_) [11] and the Boyle-Vigmond (SF_VB_) [12] definitions.

**Figure 1:**
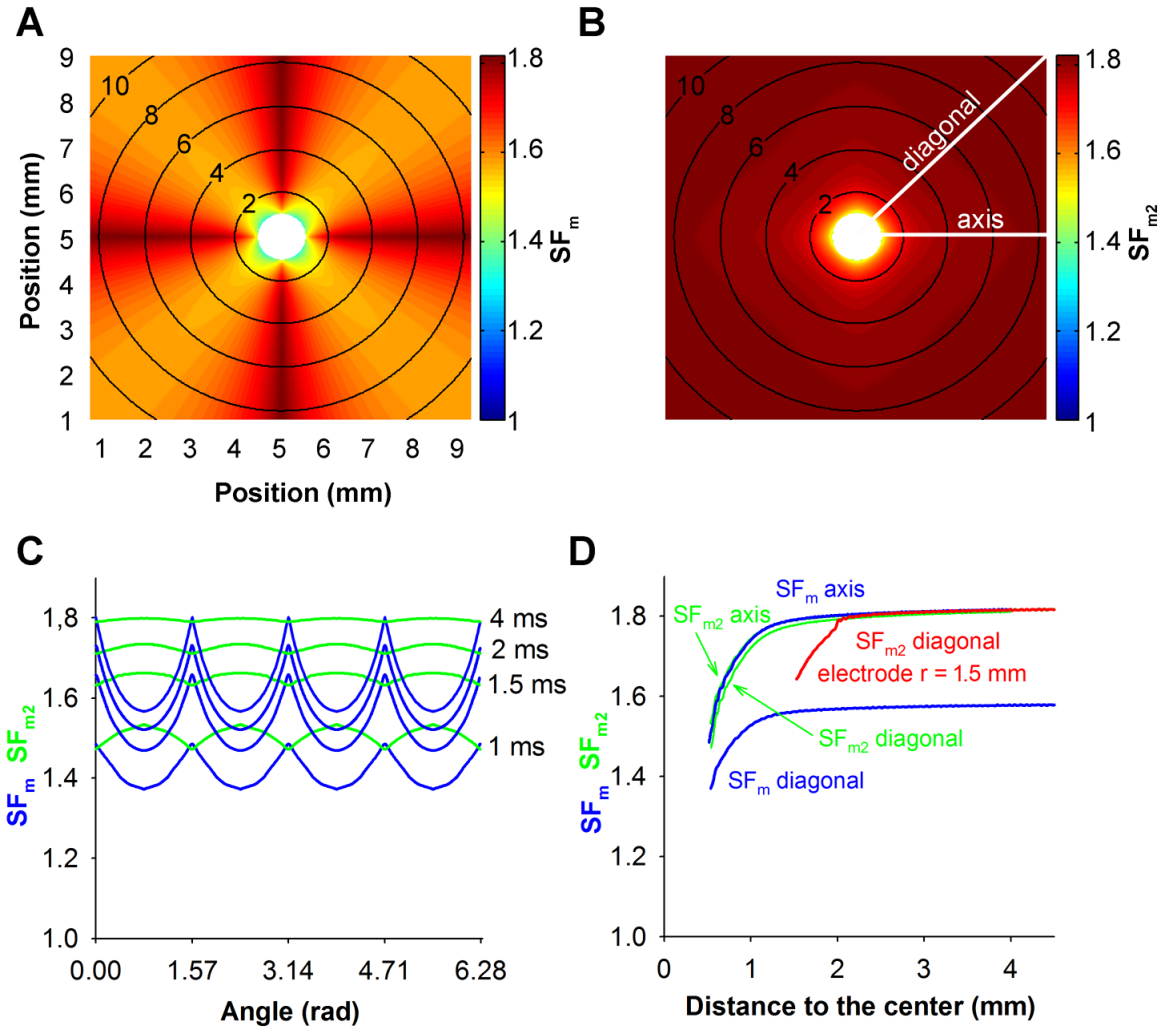
Comparison between SF_m_ and enhanced SF_m2_ formulation of the safety factor. Excitation waves were generated in an isotropic tissue stimulated with a 0.5(white colored) at the center of the tissue. A and B: Activation sequences are represented by isochrones (black lines, numbers indicate the instant of activation in ms) and the safety factor computed using SF_m_ (A) and SF_m2_ (B) is color-coded. Tissue boundaries were not shown for the sake of clarity. C: SF_m_ (blue lines) and SF_m2_ (green lines) as a function of the angular coordinate along the wavefront in the control tissue at four instants, 1 ms, 1.5 ms, 2 ms and 4 ms after the onset of the simulation. D: Evolution of the SF_m_ (blue lines) and SF_m2_ (green lines) with distance to the center of the electrode in the diagonal and in the horizontal axis of the tissue (see white lines in Figure 1B). The evolution of the SF_2m_ with distance to the center of the electrode in the diagonal of the tissue stimulated with a 1.5 mm radius circular shaped electrode has also been included (red lines).

The SF_m2_ was computed using the following equation:
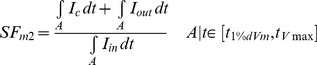
(2)where I_c_ is the capacitive current, I_out_ is the axial current that leaves the cell, I_in_ is the axial current that enters the cell and A is the integration interval, which is delimited by the instant when membrane potential derivative reaches 1% of its maximum (t_1%dVm_) and the instant of maximal V_m_ (t_Vmáx_) during the depolarization phase (see [11] for details). I_in_ and I_out_ are always positive in Eq. 2, as contributions of axial currents to I_in_ and I_out_ are considered in absolute value [11]. Computation of I_in_ and I_out_ was improved by considering the direction of these currents in the 2D tissue. As the 2D tissue was discretized using a grid, I_in_ and I_out_ were computed as the root square of the sum of the squares of their components in both Cartesian axis (see Eq. 2 and Eq. 3).
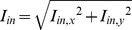
(3)

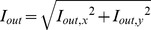
(4)This modification yields a more accurate characterization of the source-sink relationship, as illustrated in Figure 1. This figure shows the activation sequence and the characterization of the source-sink relationship in an isotropic tissue stimulated with a 0.5 mm radius circular shaped electrode at the center of the tissue (white colored) using our previous (SF_m_, Figure 1A) and our improved (SF_m2_, Figure 1B) formulation of the SF. A comparison of both SF distributions (blue lines for SF_m_ and green lines for SF_m2_) as a function of the angular coordinate along the wavefront in the control tissue at four instants, 1 ms, 1.5 ms, 2 ms and 4 ms after the onset of the simulation, which are located close to the simulation site, is also included in Figure 1C. Stimulation applied in a homogeneous isotropic tissue with the circular electrode gives rise to circular isochrones (black lines in Figures 1A and 1B, numbers indicate the instant of activation in ms). In this tissue, the expected distribution of the source-sink relationship would present a circular symmetry. When the source-sink relationship is characterized using our previous formulation (SF_m_), a dependence on the direction of propagation relative to the discretization grid was observed instead of circular symmetry (Figure 1A and blue lines in Figure 1C). However, with our improved formulation, the circular symmetry in the SF_m2_ is more faithfully preserved (Figure 1B and green lines in Figure 1C). In 1D strands both formulations SF_m2_ and SF_m_ are equivalent. It is to be noted that small differences in SF_m2_ are found along the wavefront. Figure 1C shows that the maximum difference in SF_m2_ along the wavefront is smaller than 0.5 (see the wavefront at instant 1 ms after the onset of the simulation), which is smaller than the 3% of the average of the SF value in this instant, and it is reduced as the wavefront propagates. Indeed, the maximum difference in SF_m2_ at 4 ms after the onset of the simulation is 0.008 (0.5% of the average SF_m2_ value at this instant). In the case of the SF distribution obtained with our previous SF formulation (SF_m_), the maximum difference along the represented instants is approximately 0.23 (at 4 ms) and it is not reduced as the wavefront propagates. Finally, Figure 1D shows the evolution of the SF with distance to the center of the electrode in the diagonal and in the horizontal axis of the tissue (white lines in Figure 1B) using both formulations. The curves representing the evolution of the SF_m2_ with distance to the center of the electrode in the diagonal and in the axis of the tissue are almost superimposed (green lines) as differences in SF along the wavefront are negligible, while the curves representing the SF_m_ (blue lines) are not. Therefore, our improved formulation of the SF, SF_m2_, seems appropriate to analyze the source-sink relationship in 2D tissues (see Figure S1, Figure S2 and “Additional Aspects of the Safety Factor Computation” section in File S1 for more details).

The SF_VB_ was calculated as follows:
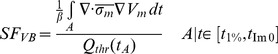
(5)

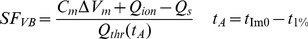
(6)where C_m_ is the membrane capacitance, Q_s_ is the intracellular stimulus charge, A is the interval from 1% take off (t_1%_) to zero membrane current (t_Im0_), during which I_m_ is positive, and Q_thr_ is the minimum charge required to elicit an AP in a single cell, which is dependent on the stimulus duration (t_A_) [12].

## Results

### Source-sink relationship of the excitation wavefront propagation

**Figure 2:**
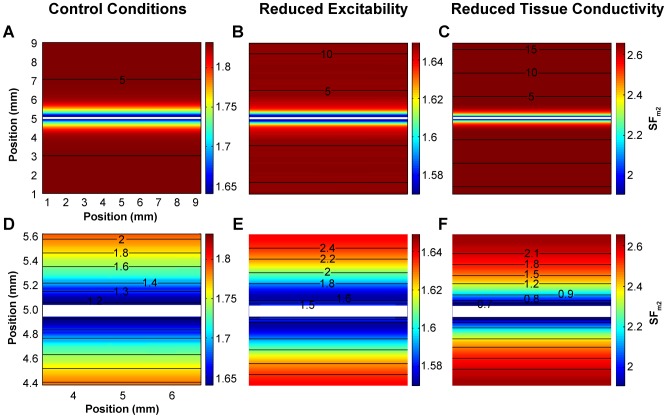
Activation sequence and distribution of the safety factor in planar waves. Excitation waves were generated in an isotropic tissue stimulated with a 10×0.1 mm electrode (white colored) at the center of the tissue that spanned the entire width of the preparation. Consequently the source-sink relationship was constant along the entire length of the electrode. Activation sequences are represented by isochrones (black lines, numbers indicate the instant of activation in ms) and the safety factor is color-coded in all panels. Bottom panels are zooms of top panels. A and D: control conditions; B and E: reduced excitability (50% reduction of the maximum sodium conductance (g_Na_)); and C and F: reduced tissue conductivity (75% tissue conductivity reduction). Tissue boundaries were not shown for the sake of clarity. Under all conditions no curvature developed during propagation.

**Figure 3:**
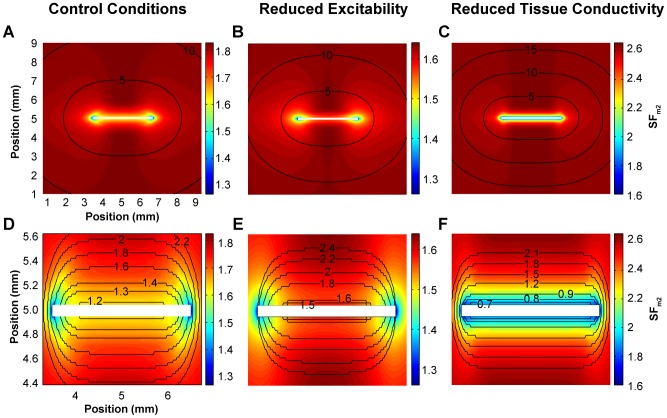
Activation sequence and distribution of the safety factor in evolving waves. Excitation waves were generated in an isotropic tissue stimulated with a 3×0.1 mm electrode (white colored) at the center of the tissue in order to observe the effect of increased load present at the ends of the electrode. The end of the electrode results in an increased load for the cells at the ends of the electrode thus creating a non-uniformity of the source-sink relationship along the length of the electrode. Activation maps are represented by isochrones (black lines, numbers indicate the instant of activation in ms) and the safety factor is color-coded in all panels. Bottom panels are zooms of top panels. A and D: control conditions; B and E: reduced excitability (50% reduction of the maximum sodium conductance (g_Na_)); and C and F: reduced tissue conductivity (75% tissue conductivity reduction). Tissue boundaries were not shown for the sake of clarity.

The activation sequence in tissue stimulated with two different electrode sizes was analyzed together with their corresponding source–sink relationship for different conditions of excitability and conductivity. Figures 2 and 3 relate the activation sequence to the distribution of the SF_m2_ in the tissue stimulated with a 10 mm×0.1 mm electrode and a 3 mm×0.1 mm electrode placed in the centre of the tissue, respectively. Top panels of both figures and their zooms (bottom panels) show the activation maps represented by isochronal lines (black lines, numbers indicate the instant of activation in ms) in control conditions, under reduced membrane excitability, and under reduced tissue conductivity from left to right, superimposed to the corresponding color-coded SF_m2_ distribution. Electrodes are white colored and the electrical activity of tissue boundaries was not shown for the sake of clarity.

As shown in Figure 2, panels A, B and C and their zooms (panels D, E and F), stimulation of the tissue with an electrode of the same length as the width of the tissue elicited planar wavefronts, as expected. All three planar wavefronts uniformly traveled from the center of the tissue to the bottom and top edges, although at different conduction velocities, as evidenced by the difference in isochronal lines densities in the top panels. This figure shows that the SF_m2_ corresponding to planar waves is uniform in the whole tissue, except at the beginning of propagation, which takes place in the region immediately surrounding the electrode. In this region (zoomed in Figure 2, panels D, E and F), the SF_m2_ evolves from the initial value registered where the propagation starts to the corresponding SF_m2_ value for planar propagation in each situation. The SF_m2_ value for planar propagation in control is 1.82 (Figure 2D). This SF_m2_ value decreases to 1.64 when the maximal sodium current conductance (g_Na_) is reduced by 50% and increases to 2.65 when the tissue conductivity is reduced by 75%. Interestingly, this figure shows that the SF_m2_ along each isochronal line is uniform.

Figure 3, panels A, B and C and their zooms (Figure 3, panels D, E and F) show the activation sequence of the propagating wave when the tissue is stimulated with a 3 mm×0.1 mm electrode located in the center of the tissue under control conditions, under decreased membrane excitability and under reduced tissue conductivity, respectively. In all these cases, the wavefront was planar at the beginning of the propagation (see the first isochronal line in Figure 3, panels D, E and F) and the SF_m2_ along the wavefront was uniform except at the ends of the stimulus electrode. In addition, the isochronal line depicted in each panel reveals that the wavefront starts to curve at each end of the wavefront. At this instant the SF_m2_ is constant in the planar part of the wavefront but a dispersion of the SF_m2_ is found where the wavefront has started to curve, the SF_m2_ being smaller in the curved region than in the planar region of the wavefront. This figure shows that a tissue stimulated with an electrode shorter than its width has a non-uniform dispersion of the SF_m2_ (i.e. the SF_m2_ along the wavefront is not uniform), differing from the SF_m2_ uniformity observed when the electrode covers the whole width of the tissue (see Figure 2). In the central part of the tissue, where the stimulus is applied, the SF_m2_ yields an initial value that rapidly rises to 1.82 in control, 1.64 under 50% g_Na_ reduction and 2.65 under 75% tissue conductivity reduction. These values correspond to the SF_m2_ for planar propagation (see Figure 2) in that specific situation as the wavefront geometry in this part of the tissue is planar. However, the SF_m2_ in the tissue close to the ends of the electrode yields the smallest values, as a result of a local increase of the electrical sink. Indeed, the local excitatory current supplied by the cells at the end of the electrode (electrical source) diverges into a large area downstream (electrical sink). As shown in Figure 3, panels D, E and F, the area of the tissue surrounding the ends of the electrode experiences a high dispersion of the SF_m2_ and matches the area where the wave curves very sharply. In addition, the local dispersion of the SF_m2_ is reduced as propagation progresses, which is related to the smoothing of the curvature. Our results reveal that the spatial distribution of the source-sink ratio of an evolving wavefront experiences a non-uniform dispersion as the SF_m2_ of the wavefront at a certain instant is not uniform. Furthermore, the areas with lower SF_m2_ show the higher wavefront curvature.

**Figure 4:**
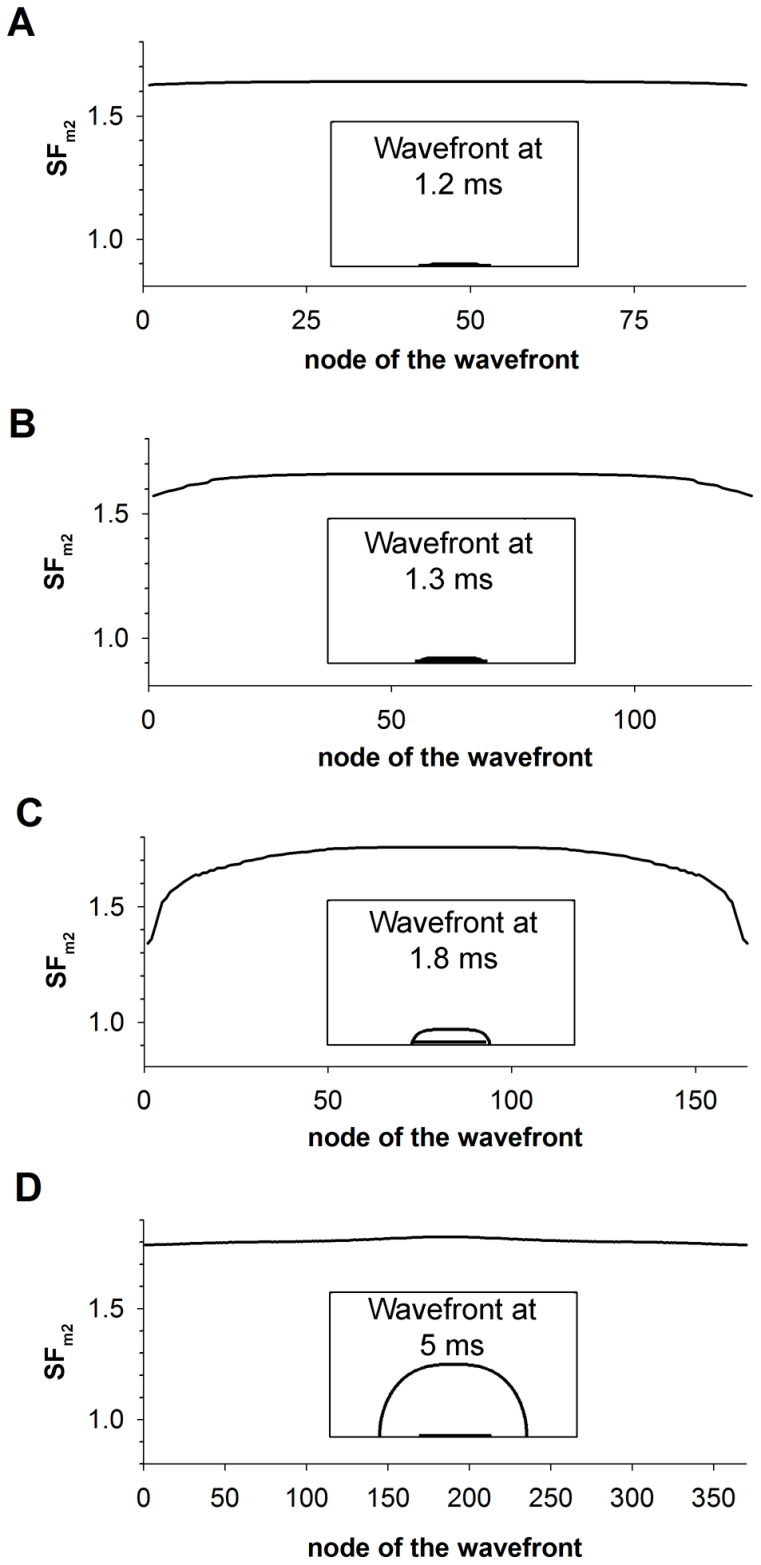
SF_m2_ along the wavefront. SF_m2_ along the symmetrical half of the wavefront propagating in the up direction in the control tissue stimulated with a 3 mm×0.1 mm electrode placed in the centre of the tissue at four instants, 1.2 ms (A), 1.3 ms (B), 1.5 (C) and 5 ms (D) after the onset on the simulation. Insets highlight the wavefront and the electrode in half of the tissue at the corresponding instants.

Figure 4 depicts the SF_m2_ along a symmetrical half of the wavefront propagating in the up direction in the control tissue stimulated with a 3 mm×0.1 mm electrode placed in the centre of the tissue at four instants, 1.2 ms, 1.3 ms, 1.8 ms and 5 ms after the onset on the simulation (panel A, B, C and D, respectively). Insets highlight the wavefront and the electrode in half of the tissue at the corresponding instants. Figure 4A shows that the SF_m2_ along the wavefront just after the initiation of the propagation is uniform, except at the ends, and the wavefront is planar, except at both ends where the SF_m2_ is slightly smaller. Figure 4B illustrates that the SF_m2_ along the wavefront decreases monotonically away from the center. Near the ends, where the wavefront curls, the SF_m2_ is reduced. Figure 4C also shows that the SF_m2_ remains non uniform as the wavefront evolves and that it is smaller where the curvature increases. Finally, Figure 4D shows that the SF_m2_ is more uniform once the wavefront of the geometry is stabilized. The SF_m2_ along approximately constant curved wavefronts is only slightly smaller than in planar wavefronts (Figure 4D). Indeed, when the curvature is not very high, cells take slightly longer to meet the sink requirement for excitation thereby by reducing the velocity of conduction.

Figure 1D shows the evolution of the SF with respect to the distance from the center of the 0.05 mm radius circular shaped electrode. This figure shows that the SF_m2_ (green lines) rapidly increases from 1.45 to 1.76 within the first 0.7 mm next to the border of the electrode and then it increases very slowly. The radius of the curvature at a source site has a strong influence on the SF_2m_. The evolution of the SF_2m_ as a function of the distance from the center of the electrode along the diagonal of the tissue when the radius of the electrode size was increased three-fold (1.5 mm) has also been included (red lines). In this case, the SF_2m_ also increases in the region surrounding the electrode and then overlaps the curve obtained with the 0.5 mm radius electrode for distances to the center longer than 2.1 mm. Consequently, our simulations reveal that the influence of the radius of curvature on the SF_m2_ is relevant when the radius is approximately smaller than 1.5 mm.

**Figure 5:**
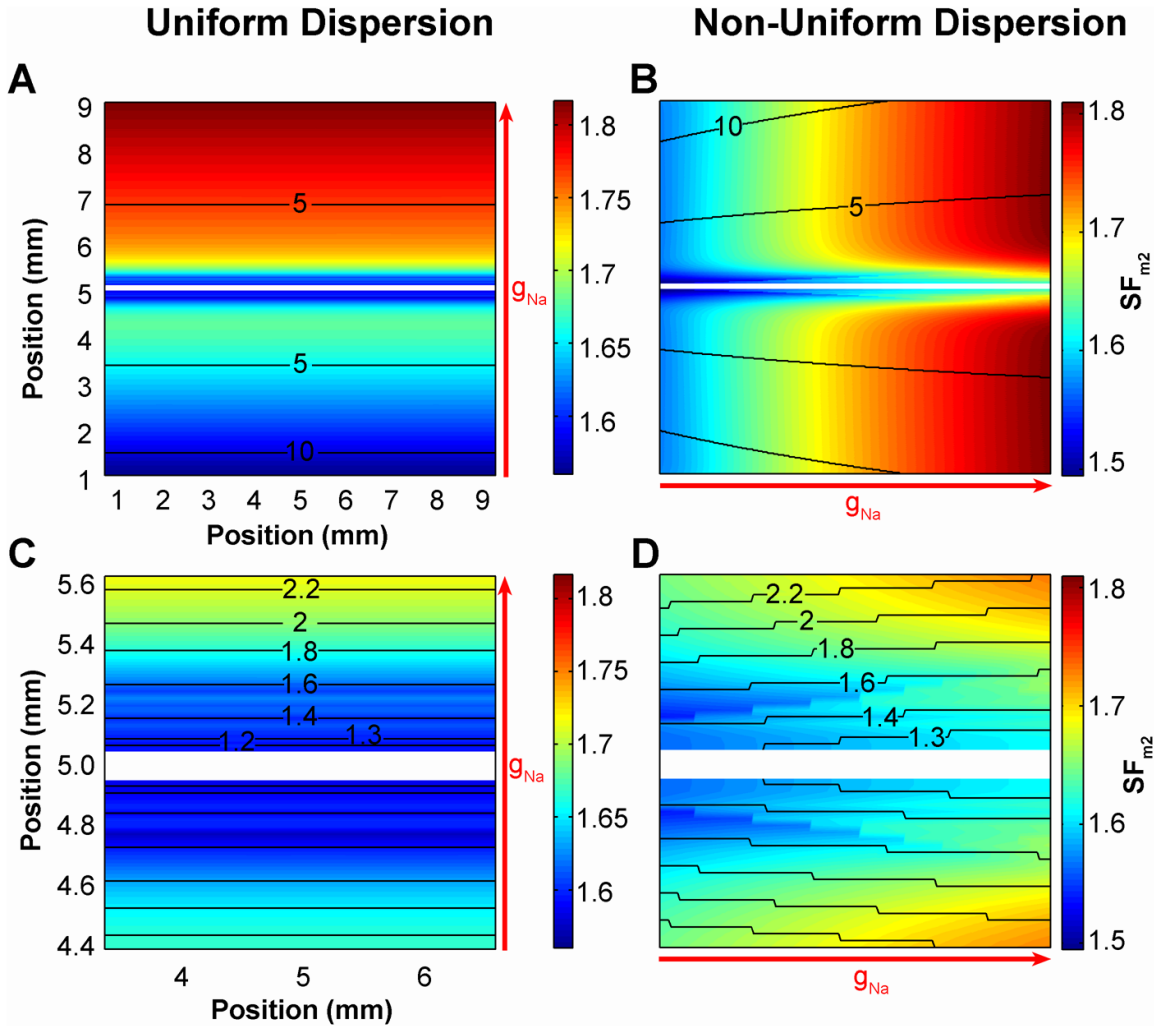
Activation sequence and distribution of the safety factor in tissue with a linear gradient of membrane excitability. Tissue with linear maximum sodium conductance (g_Na_) gradients parallel (A, C) and perpendicular (B, D) to the direction of propagation are used to explore wavefront curvature evolving in response to uniform and non-uniform dispersion of the source-sink relationship. Excitation waves were generated in an isotropic tissue with the maximum sodium conductance (g_Na_) linearly varying from 30% to 100% of the control value along the y (A and C) or x (B and D) axis (as indicated by the red arrows) stimulated with a 10 mm×0.1 mm electrode (white colored) at the center of the tissue. Activation sequences are represented by isochrones (black lines, numbers indicate the instant of activation in ms) and the safety factor is color-coded in all panels. Bottom panels are zooms of top panels. Tissue boundaries were not shown for the sake of clarity.

Our hypothesis that the spatial non-uniform distribution of the source-sink relationship along the wavefront alters the geometry of the wavefront was also tested in a tissue with a linear gradient of membrane excitability stimulated with an electrode of the same length as the width of the tissue. Figure 5, panels A and D show the activation sequence and the SF_m2_ distribution in a tissue with the maximum sodium conductance (g_Na_) linearly varying from 30% to 100% of the control value and stimulated with an electrode perpendicular to the direction of the g_Na_ gradient. In this case, the tissue elicited planar wavefronts and the SF_m2_ was uniform along the wavefront despite the existence of a dispersion of the SF_m2_ in the tissue (uniform dispersion of the SF_m2_) caused by the linear gradient of g_Na_. The geometry of the wavefront was maintained because the source-sink relationship was uniform along the wavefront. However, when the electrode was placed across the g_Na_ gradient, the SF_m2_ along the wavefront was not uniform (non-uniform dispersion of the SF_m2_) and the wave developed curvature after excitation (see the first isochronal line in Figure 5D) and evolved throughout the tissue, as shown in Figure 5, panels B and D.

These results illustrate that the source-sink ratio of an evolving wavefront can lead to a non-uniform dispersion of SF_m2_ and that non-uniform dispersion of the cellular source-sink relationship leads to alterations in curvature. Furthermore, the areas with lower source-sink ratios result in conduction slowing thereby increasing local curvature.

### Source-sink relationship at the initiation of the excitation wave

To better understand the role of the source-sink relationship in the process of wavefront evolution, wave dynamics at the initiation of excitation was analyzed together with its corresponding source–sink relationship in control conditions, under reduced membrane excitability, and under reduced tissue conductivity. This study was performed in a 1D strand for the sake of simplicity.

**Figure 6:**
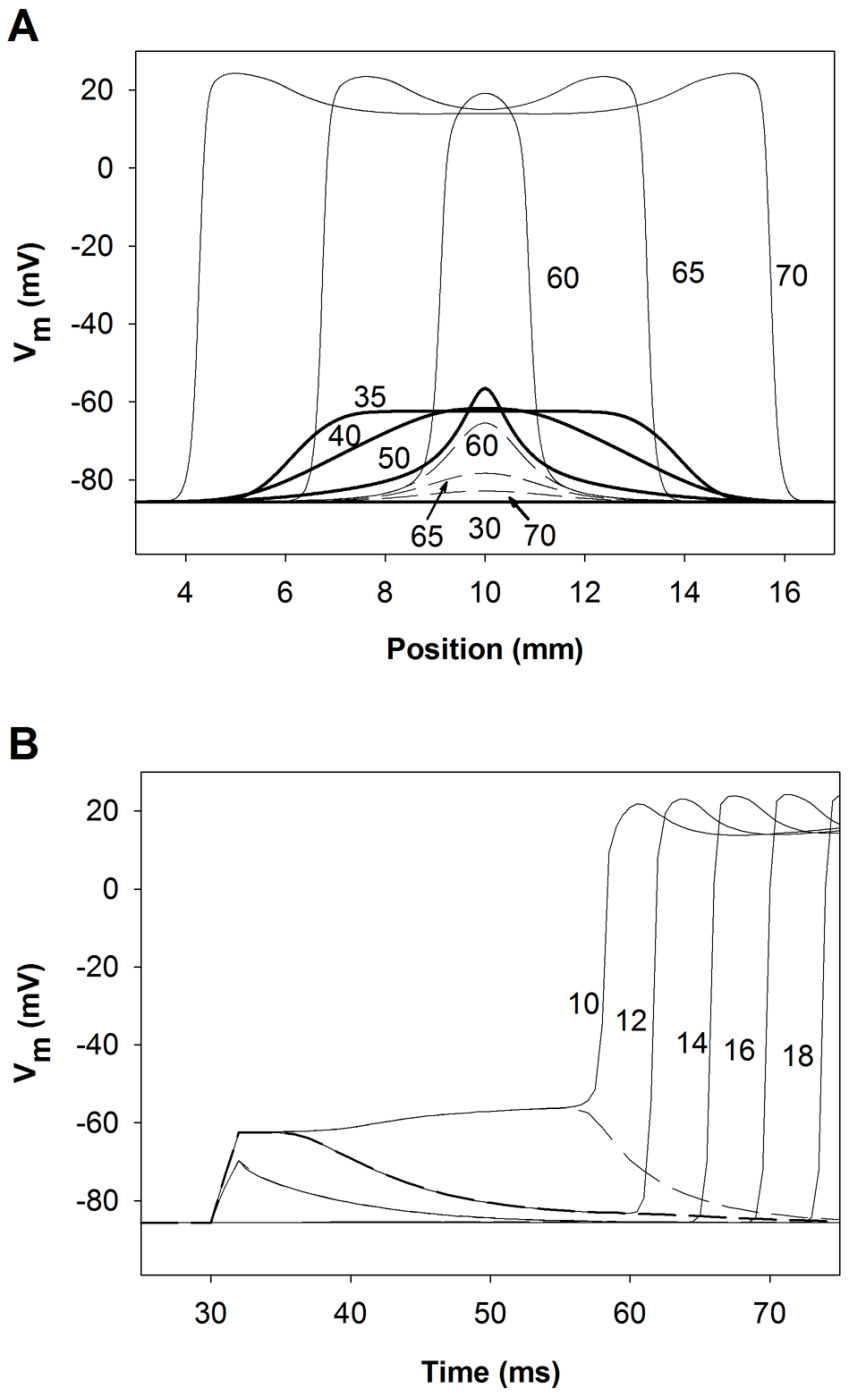
Dynamics of expanding and collapsing waves stimulated with current amplitudes very close to the threshold. Dashed and solid lines represent collapsing and expanding waves, respectively. Current amplitudes were 12.792378055655138 and 12.792378055655137 µA/µF, respectively. A: Membrane potentials along the fiber at selected instants (numbers indicate the instant in ms), thick lines represent superposed collapsing and expanding waves after current injection; and B: Membrane potential time course at selected positions (numbers indicate the position in mm).

Expanding and collapsing waves are visualized in terms of membrane potentials as a function of distance for different time instants in Figure 6A. The magnitude of the stimulation amplitude in both cases being very close to the threshold, 12.792378055655138 µA/µF (expanding front) and 12.792378055655137 µA/µF (collapsing front), respectively. High precision in the stimulation amplitude is needed to observe the electrophysiological activity close to the threshold for propagation. In addition, the leading edge of the action potential as a function of time is shown for positions 10 mm, 12 mm, 14 mm, 16 mm and 18 mm in Figure 6B. In both cases, application of the stimulus current increases membrane potential to the same extent at the point of current injection (thick lines in Figure 6A). When the stimulation is very close to the threshold, the time to either propagation or collapse can be quite long as the cellular sources engage in charge transfer to the sink region (see Figure 6B). If the amplitude of the stimulation current is higher than the threshold, the center of the fiber depolarizes giving rise to the formation of the excitatory wave (see solid lines in Figure 6, panels A and B), that expands along the fiber. Conversely, if the stimulus amplitude is smaller than threshold, membrane potential in the central region rises until the sink demands cannot be met. Subsequently, the membrane potential decreases, signaling a collapse of the wave (see dashed lines in Figure 6, panels A and B).

**Figure 7:**
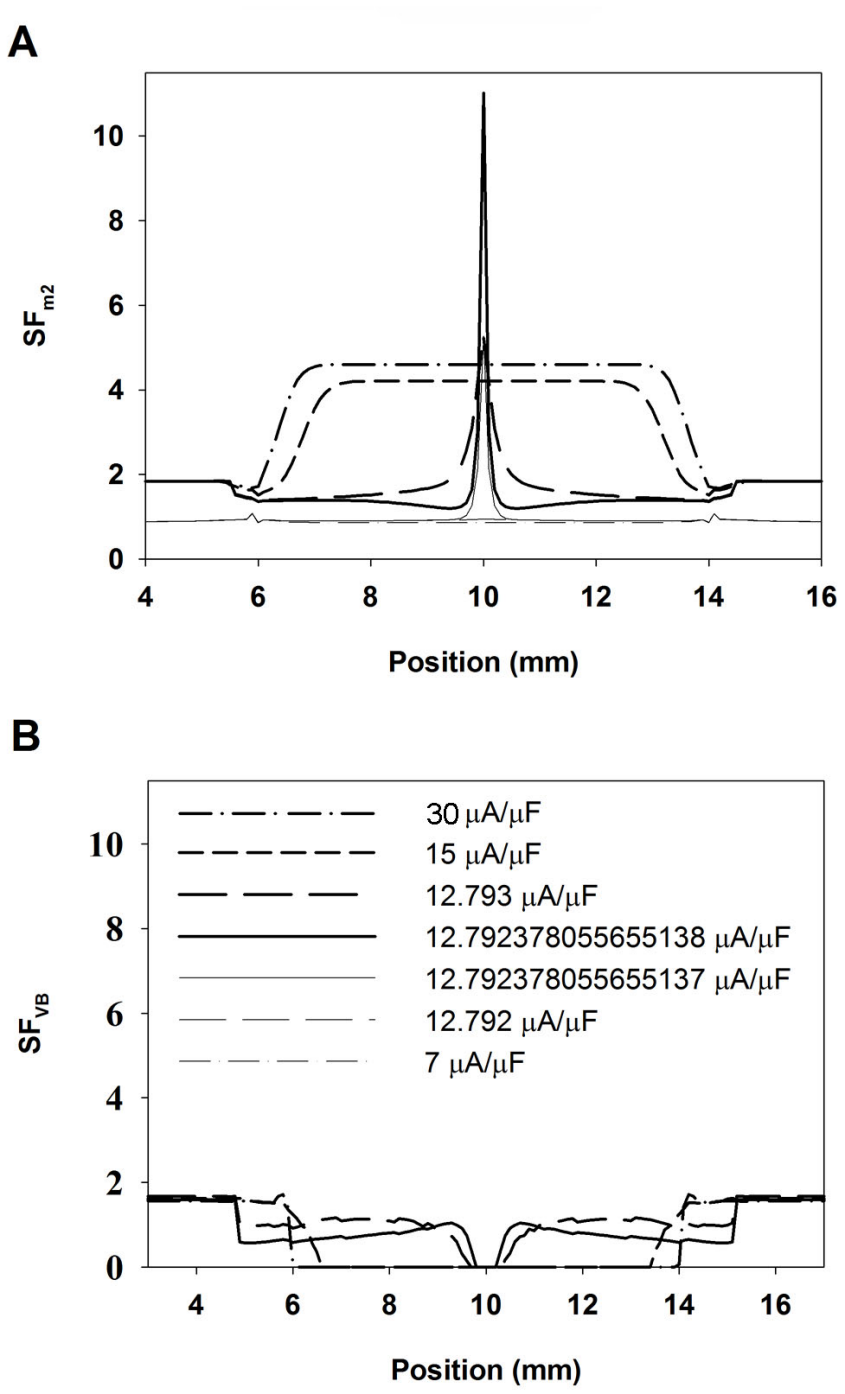
Safety factor profiles of expanding (thick lines) and collapsing (thin lines) waves. A: Safety factor profiles obtained using our safety factor formulation (SF_m2_); and B: safety factor profiles obtained using the safety factor formulation proposed by Boyle and Vigmond (SF_VB_). Waves were produced with different stimulation amplitudes (30 and 7 (dotted lines), 15 (short dashed lines), 12.793 and 12.792 (long dashed lines), 12.792378055655138 and 12.792378055655137 (solid lines) µA/µF, as indicated in panel B).

The characterization of the source-sink relationship in the control 1D strand for different stimulation amplitudes is depicted in Figure 7. Figure 7, panels A and B show the SF distributions obtained using the improved version of the Romero et al. formulation (SF_m2_) and the Boyle-Vigmond formulation (SF_VB_), respectively. Figure 7A shows that expanding waves, resulting from suprathreshold stimuli have SF_m2_ values (thick lines) greater than unity throughout the strand, the specific value of the SF_m2_ depending on the wavefront location and the stimulation amplitude. Suprathreshold stimulus amplitudes of 30 and 15 µA/µF, which are approximately 2.5 and 1.25-fold the threshold amplitude, generate expanding waves with SF_m2_ values of 4.5 and 3.8, respectively at the beginning of the propagation. The decline of the SF_m2_ with the reduction of the stimulation amplitude is a consequence of the decrease of the source charge available to sustain the propagation process. As both waves approach the ends of the electrode the value of the SF_m2_ markedly decreases, reaching its minimum (1.6) at the ends of the electrode. The SF_m2_ fall at both ends of the electrode is provoked by the increase of the electrical load that reflects that portion of the fiber that is not directly stimulated by the electrode. As the stimulation amplitude decreases approaching the threshold, the SF_m2_ in the region directly stimulated by the electrode is reduced except in the central zone of the electrode where the SF_m2_ increases very sharply (thick solid line). Note that the region where the SF_m2_ rises matches very well the region where the excitatory wave initiates. As the expanding waves propagate away from the ends of the electrode the SF_m2_ rises to reach a value of 1.84, which is similar to the SF_m2_ of the planar wave in the 2D control tissue (Figure 2D). Conversely, subthreshold stimulation produces collapsing waves and the SF_m2_ is smaller than unity in most of the strand (Figure 7B, thin lines). When the strand is stimulated with a current of 7 µA/µF amplitude, the SF_m2_ is smaller than unity throughout the strand except at the end of the electrode where a small oscillation is observed. As the subthreshold stimulation approaches the threshold, a local increase of the SF_m2_ in the center of the fiber is observed, but SF_m2_ values associated with the collapsing wave are always smaller than the SF_m2_ observed when the suprathreshold stimulation is close to the threshold. This observation suggests that a critical source-sink relationship separates wave expansion from wave collapse and its characterization along the strand would be delimited by the SF_m2_ profiles obtained for the highest subthreshold and the smallest suprathreshold stimulation amplitude. Figure 7B shows that the magnitude of the SF_VB_ is not greater than unity throughout the strand when it is stimulated with current amplitudes higher than the threshold (thick lines). Suprathreshold stimulus amplitudes of 30 and 15 µA/µF (2.5 and 1.25-fold the threshold magnitude, approximately) result in expanding waves, in conflict with SF_VB_ which is equal to zero in most of the region directly stimulated by the electrode. Subsequently, the SF_VB_ increases as the wave approximates the electrode and remains constant (1.6 approximately) when the wave travels far from the electrode. As the stimulus amplitude is reduced to near threshold, the SF_VB_ increases in the region directly stimulated by the electrode. In the area where the propagating wave is generated the SF_VB_ is zero. The SF_VB_ is zero in the region where the propagating wave is generated because I_m_ is negative during the depolarization phase, which cancels the interval during which the charge delivered to the cell is computed. Subthreshold stimulation results in a SF_VB_ equal to zero throughout the strand. The aforementioned results suggest that the SF_VB_ incompletely characterizes the source-sink relationship at the initiation of the excitation wave. Long regions of the strand have a SF_VB_ value smaller than unity when suprathreshold stimulation is applied and the zone where the wave is initiated displays the minimum SF_VB_ value (zero). Furthermore, an increase of the stimulus amplitude (source) does not raise the SF_VB_ in the zone of stimulation. Therefore, the SF_m2_ formulation is preferable for characterizing the source-sink relationship. The use of the SF_m2_ to characterize the source sink relationship at the moment of excitation and development of the propagating wave reveals a critical source-sink relationship distribution, separatrix, that separates expanding waves from collapsing waves.

### Influence of electrode length, membrane excitability and tissue conductivity on the source-sink relationship at the initiation of the excitation wave

**Figure 8:**
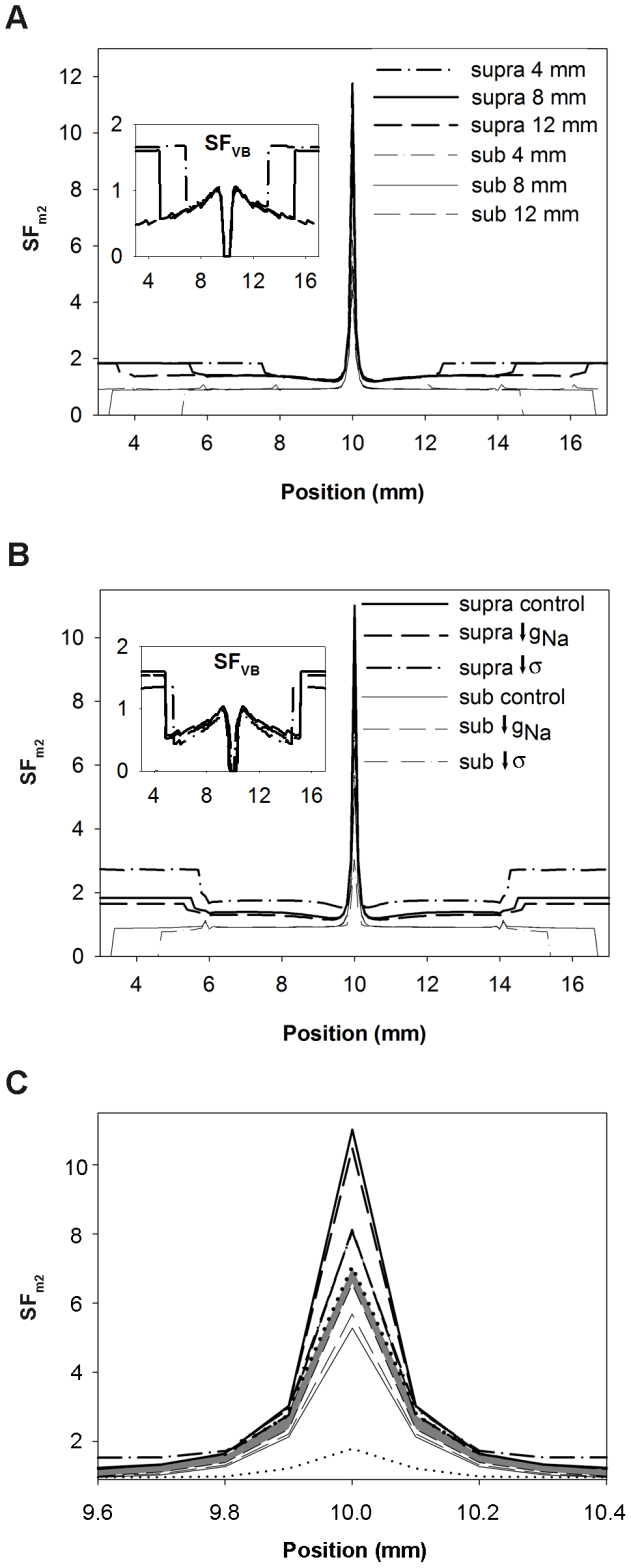
Effect of the electrode length, membrane excitability and tissue conductivity on the safety factor. Safety factor profiles obtained with the safety factor formulation SF_m2_ for suprathreshold (thick lines) and subthreshold (thin lines) amplitudes close to the threshold are represented. A: 4 mm (dashed dotted lines), 8 mm (solid lines) and 12 mm (dashed lines) electrode size, the inset shows the SF_VB_ profiles for comparison; B: control (solid lines), 50% reduction of the maximum sodium conductance (g_Na_) (dashed lines) and 75% reduction of tissue conductivity (σ) (dashed dotted lines), the inset shows the SF_VB_ profiles for comparison; and C: control (solid lines), 50% reduction of the maximum sodium conductance (g_Na_) (dashed lines) and 75% reduction of tissue conductivity (dashed dotted lines), 70% reduction of g_Na_ (short dashed lines) and 87.5% reduction of tissue conductivity (dotted lines). The shaded profile indicates the critical safety factor obtained with our formulation (SF_m2_).

The influence of the electrode length and the active and passive properties of the tissue at the initiation of the propagating wave in the 1D strand were analyzed using the SF_m2_ formulation. Our results are summarized in Figure 8.

Figure 8A depicts the SF_m2_ in the 1D strand when it was stimulated with suprathreshold (thick lines) and subthreshold (thin lines) amplitudes very close to the threshold for three different electrode lengths: 4 mm (dashed dotted lines), 8 mm (solid lines) and 12 mm (dashed lines). This figure shows that the SF_m2_ profiles of the waves elicited by these three electrodes were very similar. In all cases, the SF_m2_ experienced a marked increase where the wave initiates, it sharply changed in the region surrounding both electrode ends and the SF_m2_ of expanding waves reached the value 1.8 far from the electrode. Inset of Figure 8A shows the SF_VB_ profiles for comparison. In this case, the SF_VB_ profiles elicited by these three electrodes are very similar. The SF_VB_ is zero in the region where the wave is generated and resembles the profiles shown in Figure 7B. Figure 8B shows that a 50% reduction of g_Na_ (dashed lines) produced an overall reduction of the SF_m2_ value throughout the strand with respect to control (solid line), while a 75% reduction of tissue conductivity (dashed dotted lines) resulted on an increase of the SF_m2_ except in the center of the fiber. Inset of Figure 8B shows the SF_VB_ profiles under these conditions. A 50% reduction of g_Na_ (dashed lines) also reduced the SF_VB_. However, a 75% reduction of tissue conductivity (dashed dotted lines) reduced the SF_VB_, in contrast to the increase of the SF_m2_ observed in this situation. Superposition of the SF_m2_ profiles obtained when the stimulation was the closest to the threshold as possible in all the simulated conditions showed that the values of the SF_m2_ of the expanding waves were higher than the values for the collapsing waves in every position of the strand (Figure 8B), which is not observed when the SF_VB_ is considered. This suggests that a unique SF_m2_ profile would characterize the limit between propagation and failure, regardless of the excitation electrode size, the membrane excitability and the tissue conductivity. This critical SF_m2_ profile would be delimited at each node of the strand by the maximum SF_m2_ value observed in that particular node for all the collapsing waves and the minimum SF_m2_ observed in that particular node for the expanding waves generated with the smallest amplitude stimulus in each condition. In order to more accurately characterize the critical SF_m2_ profile, more severe conditions, such as 70% g_Na_ reduction (short dashed lines in Figure 8C) and 87.5% tissue conductivity decrease (dotted lines in Figure 8C) were simulated. Figure 8C shows that the critical SF_m2_ value, indicated by the shaded profile, would be comprised between 6.6 (maximum SF_m2_ obtained for subthreshold stimulation) and 7 (minimum SF_m2_ peak obtained for suprathreshold stimulation very close to the threshold) in the center of the strand, which corresponds to the central point of the region where the wave is generated. Then, the critical SF_m2_ would move between 2.8 and 2.4, 1.6 and 1.4, 1.3 and 1.1, 1.2 and 1, and so on with successive 0.1 mm displacements to each side. Note that to initiate an expanding wave, the critical profile shows that a region of the tissue near the center must have SF_m2_ values above the unity. The existence of a region smaller than the critical region with high SF_m2_ values would not promote the generation of expanding waves. To summarize, the use of the SF_m2_ at the initiation of the excitation wave has allowed the characterization of the critical source-sink relationship distribution for the generation of excitation waves. This critical region was insensitive to the electrode size of excitation, the membrane excitability or the tissue conductivity.

## Discussion

### Main findings

In this study, we explored the role of uniform and non-uniform source-sink relationships on the evolution of wavefront geometry using computer simulations. An improved version of the Romero et al. safety factor formulation (SF_m2_) was developed. We related the wavefront geometry of excitatory waves to the source-sink relationship, characterized by the SF_m2_, in a 2D isotropic tissue in the absence of anatomical or functional obstacles. Our results showed that non-uniform dispersion of the source-sink relationship alters wavefront curvature. To our knowledge, this is the first time that the source-sink relationship has been characterized to gain insights about its role in evolution of the wave front geometry.

Another novel aspect of this work is the characterization of the source-sink relationship at the beginning of excitation waves. Previous studies have analyzed the source-sink relationship in waves that were already propagating. The study of the SF_m2_ in the region where currents very close to the threshold were applied has enabled not only the calibration of the two recent SF formulations but also the characterization of the critical source-sink relationship that separates expanding from collapsing waves, regardless the stimulation electrode length, the membrane excitability, and the tissue conductivity.

Our study reinforces the idea that the SF_m2_ represents a powerful tool to study the mechanisms responsible for cardiac propagation including wave initiation and evolution of wavefront curvature, providing a better understanding of cardiac arrhythmogenesis and treatment.

### Role of the source-sink relationship on wavefront curvature

Although the characterization of the source-sink relationship has been rarely documented, the source-sink approach has been widely used to explain many aspects of cardiac propagation, including the effects of curvature on cardiac propagation. For example, a convex wavefront propagates slower than a planar wavefront because the local excitatory current supplied by the cells at the front of the convex wave diffuses into a larger downstream area [2]. In addition, the concept of critical curvature for cardiac propagation relies on the balance of local currents or the ratio between sources and sinks [1], [2]. However, this is the first time that the source-sink ratio has been analyzed in order to gain new insights into the evolution of wavefront geometry. Wavefront curvature has been traditionally explained as the deviation of waves at either functional or structural obstacles [2], [3], [19], [20], but it is far from explaining the ultimate biophysical principle that governs evolution of wavefront geometry. In this paper, we demonstrate that non-uniform dispersion of the source-sink relationship alters wavefront curvature even in the absence of structural and functional obstacles. In addition, our observations are in agreement with the traditional perspective, as functional and structural obstacles may effectively alter the source-sink relationship in a non-uniform manner [9], [11].

We have also investigated the source-sink relationship in an anisotropic tissue. Figure S3 shows the activation sequence and the distribution of the safety factor in an anisotropic tissue stimulated with a 0.5 mm radius circular shaped electrode. This figure shows that when the source-sink relationship varies along the wavefront, its geometry is altered and the wave front is more curved where the SF_m2_ is smaller. Anisotropy is another mechanism for altering the source-sink relationship and extending our results to anisotropic tissue follows from the g_Na_ gradient exercise. Therefore, the non-uniform distribution of the source-sink relationship along the wavefront also alters the geometry of the wavefront in anisotropic tissues and the areas with lower source-sink ratios result in conduction slowing leading to higher wavefront curvature. Moreover, we found that uniform dispersion of the source-sink relationship does not promote wavefront curvature. A linear spatial gradient of membrane excitability in the same direction as a planar wavefront provides a substrate for uniform dispersion of the source-sink relationship in the tissue but such a structure does not promote wavefront curvature (Figure 5, panels A and C).

### The safety factor approach for studying cardiac propagation

Different parameters have been used to study cardiac propagation and associated arrhythmogenesis, such as the liminal length for wave initiation [21], which has been used to characterize the cardiac vulnerable period [22]. Moreover, the cardiac vulnerable period has been also analyzed to assess the effect of drugs [22] and mutations [23] on sodium channels. Similar to the liminal length, the liminal area was defined for 2D tissues and, used to study cardiac conduction block at a narrow isthmus [1]. In addition, spiral wave drift has been explored in the setting of a critical curvature [2], [6]. Dispersion of refractoriness has been related to vulnerability to reentry and arrhythmic episodes [3], [24], [25] [24], [26]. Unfortunately, the use of the aforementioned parameters on the study of cardiac arrhythmias presents some drawbacks as they are focused only on certain aspects of the propagation process. The SF evaluation takes into account the effect of every parameter influencing the propagation process, as it characterizes the source-sink relationship. The study of the SF_m2_ of waves elicited by stimulation currents very close to the threshold under different simulation conditions has revealed the existence of a unique critical source-sink relationship that separates collapsing waves from expanding ones, independent of the electrode size, the membrane excitability and the cellular coupling. Conversely, the liminal length, liminal area, critical curvature and refractory period depend on the active and passive properties of the tissue as well as the stimulation characteristics [1], [2], [21]. In this work, we have also shown that propagation of sharply curved wavefronts in tissues with reduced conductivity may be safer than conduction of planar wavefronts in control conditions, which could be counterintuitive. Therefore, the use of SF_m2_ can provide a better understanding of the mechanisms of arrhythmogenesis and make a useful contribution to the development of cardiac therapies.

In a previous work, we showed that the reduction of the source–sink ratio, rather than solely refractoriness, was the ultimate cause of the unidirectional block leading to reentry during regional acute myocardial ischemia [11]. Other theoretical works have also used the SF to study cardiac propagation and block. Indeed, the SF has been used to gain insights into the mechanisms of conduction on very slow propagation during reduced excitability and decreased gap junction coupling [8], to determine the kinetics and contributions to the propagation process of I_Na_ and I_CaL_ in the heterogeneous regions [9], to study the importance of obstacle composition and geometry in wavefront interactions with cardiac obstacles [14], to analyze the SF for cardiac propagation in cases of branching [13], in PVJ [12], [15], [16] or in the atrial pectinate muscle [27] and to study the effects of the increase of the effective interstitial resistivity [17].

Some experimental studies have tried to infer the effects of several factors relevant for cardiac propagation on the source-sink relationship although without characterizing the source-sink relationship [1], [28]–[30]. To the best of our knowledge no experimental measurement of the SF_m2_ has been conducted. The experimental measurement of axial currents flowing between adjacent cells that is needed to compute I_in_ and I_out_ represents a technical challenge.

### SF computation

Shaw and Rudy defined the SF of a cell in a fiber as the ratio of charge generated to charge consumed during the excitation cycle, the fraction of SF below unity indicating the margin of safety [8]. That SF formulation characterized the source-sink relationship more faithfully than Delgado and colleagues [7] and Leon and Roberge formulations [31]. Specifically, Shaw and Rudy SF decreased with membrane excitability reduction and dropped below unity when propagation failed while the other formulations did not. Our group optimized the calculation of the interval corresponding to the excitation cycle in order to facilitate its use in inhomogeneous two-dimensional tissues [10]. In the present paper, we introduce an improvement in the formulation of the SF_m_ for 2D tissues, the SF_m2_, which is independent of the direction of propagation relative to the discretization grid of the tissue. The SF_m2_ values obtained in our simulations are in line with other simulation studies. Indeed, the SF_m2_ value of 1.82 obtained for a planar wavefront propagating in control conditions is similar to the corresponding value shown in our previous work (1.6) [11] and to other SF values computed in 1D strands in control conditions [8], [9]. The difference between these values is that in our previous work membrane kinetics were simulated using a modified version of the Luo-Rudy dynamic model [32] while in this work we used the ten Tusscher and Panfilov model [18]. In addition, a 50% g_Na_ reduction decreased the SF_m2_ from 1.86 to 1.64, which is similar to the decrement from 1.6 to 1.4 observed with the Luo-Rudy dynamic model and an 87.5% decrease of the tissue conductivity produced an increase from 1.82 to 2.65, similarly to the increase from 1.6 to 2.7 observed with the Luo-Rudy dynamic model. The slowing of conduction with the reduction of membrane excitability and tissue conductivity is consistent with experimental observations (see Figure 2 and Figure 3, panels A, B and C) [7], [33].

In this work, we also demonstrate the validity of the SF_m2_ to characterize the source-sink relationship at the initiation of the excitation wave. Expanding waves have SF_m2_ values (thick lines in Figures 7A and 8) greater than unity throughout the strand, the region where the excitatory wave initiates having the higher SF_m2_ values while both ends of the electrode yield the minimum values due to the added load of cells surrounding the ends of the electrode. In addition, the SF_m2_ in the region directly stimulated by the electrode shows an overall decline with the reduction of the stimulation amplitude. Moreover, the SF_m2_ of collapsing waves is smaller than unity in most of the strand and the SF_m2_ values of collapsing waves in every strand node being always smaller than the SF_m2_ observed when the suprathreshold stimulation is close to the threshold. Finally, the study of the SF_m2_ of the response to stimulation currents very close to the threshold under different simulation conditions has revealed the existence of a unique critical source-sink relationship distribution that separates collapsing from expanding waves, regardless of the electrode size, the membrane excitability or the tissue coupling.

Boyle and Vigmond have recently proposed an intuitive formulation based on the surplus of charge delivered relative to the minimum required to trigger an action potential, which was calculated as the minimum charge required for eliciting an AP in a single cell [12]. The SF_VB_ showed an overall appropriate behavior in the PVJ, except for the increase of the SF_VB_ in Purkinje cells close to the myocardium when Purkinje strand widths were wider than 120 µm [12]. Our results show that this definition also fails to characterize the source-sink relationship at the initiation of excitation waves and under moderate tissue uncoupling, situations that were not tested by the authors. Indeed, the ratio between the charge delivered to the cell and the threshold charge (Q_thr_) of an isolated cell is not a good indicator of the source-sink relationship, as the behavior of an isolated cell is different from the behavior of coupled cells in a tissue [8]. The formulation proposed by Delgado and coworkers was also based on the margin of extra charge provided to the cell after reaching the threshold [7] and it also failed to accurately characterize the source-sink relationship [8].

To sum up, the SF_m2_ faithfully characterizes the source-sink relationship in 1D and 2D tissues, not only when the wave is propagating but also at the beginning of propagation. Therefore, the SF_m2_ is a very valuable tool for the study of the mechanisms of wave initiation and propagation, which will improve our knowledge about cardiac arrhythmias and their therapies.

### Limitations of the study

The implementation of the AP model equations and the reaction-diffusive equation using double precision variables limited the threshold amplitude currents precision to 10^−15^ µA/µF. Higher precision variables would allow us to stimulate the 1D strand with current amplitudes even closer to the threshold and, therefore, to more accurately characterize the critical source-sink relationship for wave propagation.

Finally, a 3D bidomain model would be required to reproduce the virtual electrode effect produced by electric stimulation in the vicinity of the electrode. This is crucial for simulating defibrillation shocks [34], when high currents are involved. In the present study, low current excitation was applied to stimulate the tissue and strand, which reduces the virtual electrode effect [35].

## Supporting Information

Figure S1 **Activation sequence and distribution of the safety factor in circular waves.** Waves were generated in an isotropic tissue stimulated with a 0.5 mm radius circular shaped electrode at the center of the tissue. Safety factor profiles were computed using a version of the SF computation that takes into account the inclination of the axial currents with the direction of propagation (SF_m3_). A: SF_m3_ using the direction of propagation theoretically computed (SF_m3T_). B: SF_m3_ using the direction of the propagation defined as the unitary gradient of the activation time (SF_m3AT_). Activation maps are represented by isochrones (black lines, numbers indicate the instant of activation in ms) and the safety factor is color-coded in all panels. Tissue boundaries were not shown for the sake of clarity. C: SF_m3T_ (black lines), SF_m3AT_ (red lines) and SF_m2_ (green lines) distributions as a function of the angular coordinate along the wavefront in the control tissue at four instants, 1 ms, 1.5 ms, 2 ms and 4 ms after the onset of the simulation.
(TIF)

Figure S2 **Horizontal component of the unitary vector of the direction of the propagation.** The tissue was stimulated with a 0.5 mm radius circular shaped electrode. A: Theoretically computed. B: Computed from the activation sequence.
(TIF)

Figure S3 **Activation sequence and distribution of the safety factor in an anisotropic tissue.** Waves were generated in an anisotropic tissue stimulated with a 0.5 mm radius circular shaped electrode at the center of the tissue. Activation maps are represented by isochrones (black lines, numbers indicate the instant of activation in ms) and the safety factor is color-coded. Tissue boundaries were not shown for the sake of clarity. This figure also shows that when the source-sink relationship varies along the wavefront its geometry is altered and the wave front is more curved where the SF_m2_ is smaller.
(TIF)

File S1 **Safety Factor Supplementary Material.** Safety Factor Computation Details, Additional Aspects of the Safety Factor Computation and Distribution of the Safety Factor in Anisotropic Tissues.
(DOCX)
