# Supplementary figures and images for: Non-Uniform Dispersion of the Source-Sink Relationship Alters Wavefront Curvature
Source: PLoS One. 2013 Nov 4;8(11):e78328. doi: 10.1371/journal.pone.0078328 (PMC3817246; doi:10.1371/journal.pone.0078328)

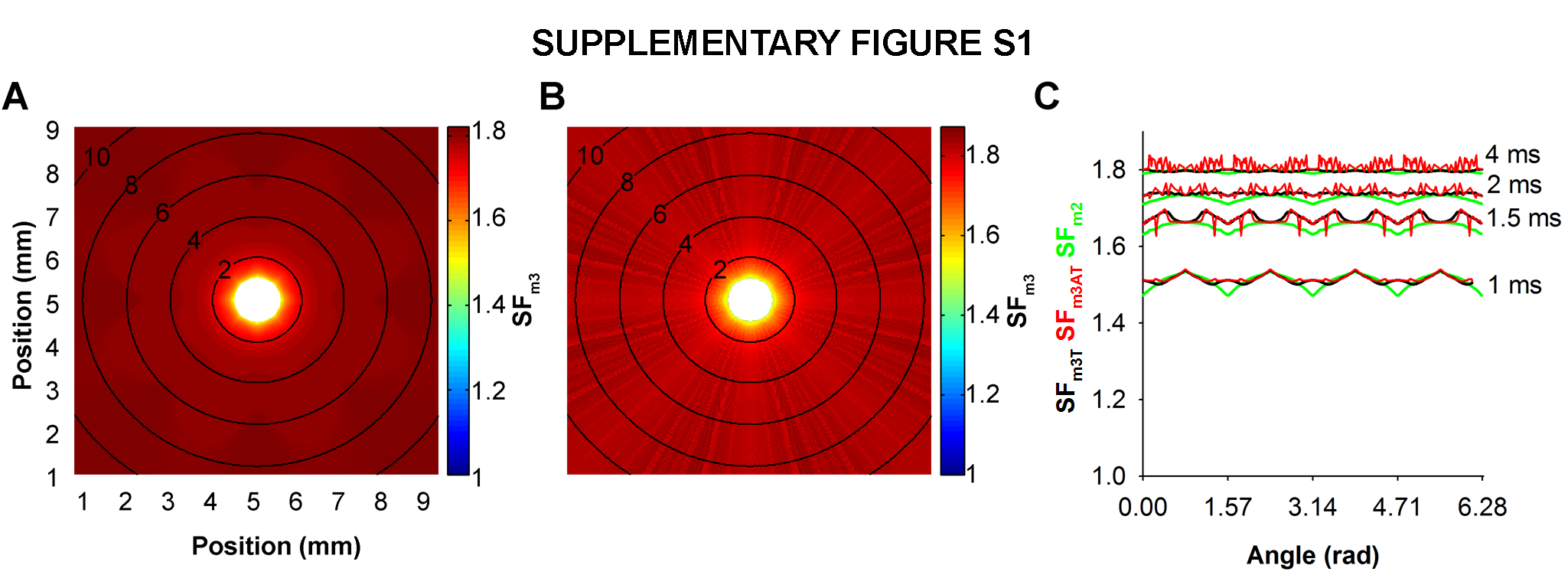

Supplement: Figure S1 — Activation sequence and distribution of the safety factor in circular waves. Waves were generated in an isotropic tissue stimulated with a 0.5 mm radius circular shaped electrode at the center of the tissue. Safety factor profiles were computed using a version of the SF computation that takes into account the inclination of the axial currents with the direction of propagation (SFm3). A: SFm3 using the direction of propagation theoretically computed (SFm3T). B: SFm3 using the direction of the propagation defined as the unitary gradient of the activation time (SFm3AT). Activation maps are represented by isochrones (black lines, numbers indicate the instant of activation in ms) and the safety factor is color-coded in all panels. Tissue boundaries were not shown for the sake of clarity. C: SFm3T (black lines), SFm3AT (red lines) and SFm2 (green lines) distributions as a function of the angular coordinate along the wavefront in the control tissue at four instants, 1 ms, 1.5 ms, 2 ms and 4 ms after the onset of the simulation. (TIF) [file pone.0078328.s001.tif]

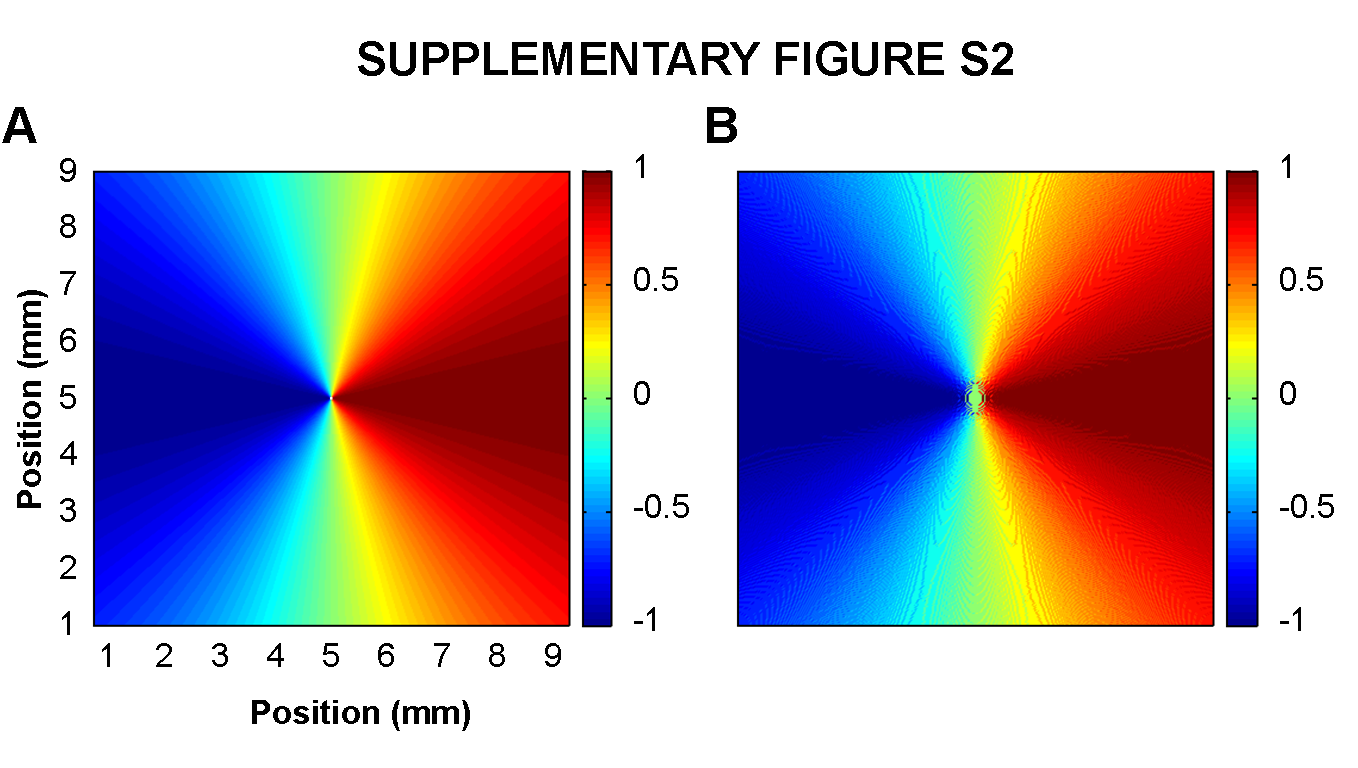

Supplement: Figure S2 — Horizontal component of the unitary vector of the direction of the propagation. The tissue was stimulated with a 0.5 mm radius circular shaped electrode. A: Theoretically computed. B: Computed from the activation sequence. (TIF) [file pone.0078328.s002.tif]

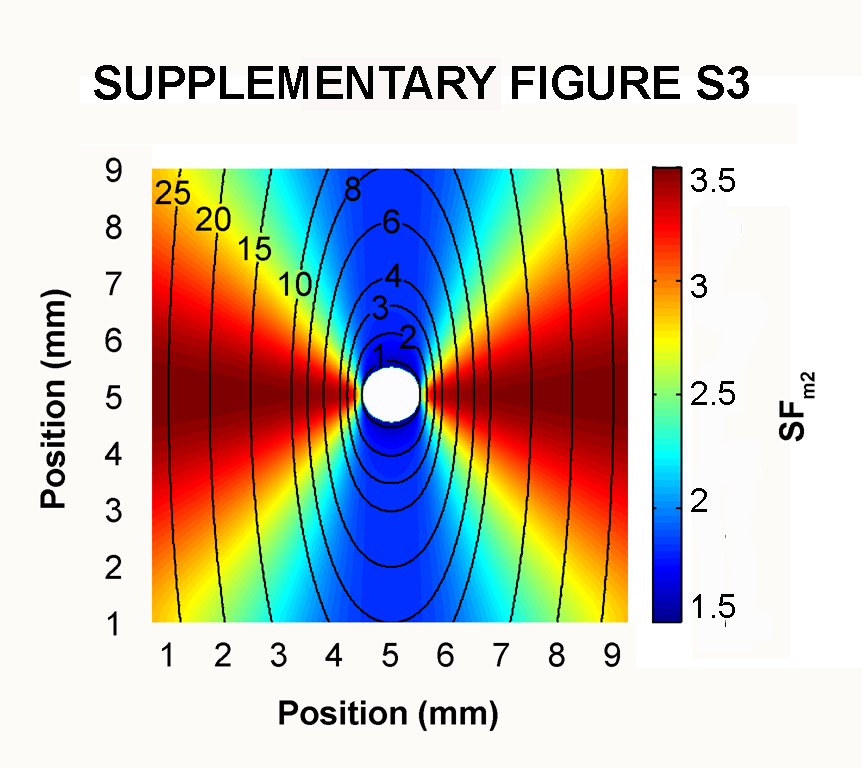

Supplement: Figure S3 — Activation sequence and distribution of the safety factor in an anisotropic tissue. Waves were generated in an anisotropic tissue stimulated with a 0.5 mm radius circular shaped electrode at the center of the tissue. Activation maps are represented by isochrones (black lines, numbers indicate the instant of activation in ms) and the safety factor is color-coded. Tissue boundaries were not shown for the sake of clarity. This figure also shows that when the source-sink relationship varies along the wavefront its geometry is altered and the wave front is more curved where the SFm2 is smaller. (TIF) [file pone.0078328.s003.tif]
