# Supplementary material for: Non-Uniform Dispersion of the Source-Sink Relationship Alters Wavefront Curvature
Source: PLoS One. 2013 Nov 4;8(11):e78328. doi: 10.1371/journal.pone.0078328 (PMC3817246; doi:10.1371/journal.pone.0078328)
Supplement: File S1 — Safety Factor Supplementary Material. Safety Factor Computation Details, Additional Aspects of the Safety Factor Computation and Distribution of the Safety Factor in Anisotropic Tissues. (DOCX) [file pone.0078328.s004.docx]

**Safety Factor Computation Details**

In this work, the units of I_c_ and the axial currents (I) are A/F, which is equivalent to V/s. These currents were computed as follows:

I_c_=dV/dt (A/F)

I=0.001*ΔV/Rtotal

Rtotal=dx/sgm

Where sgm is introduced in s^-1^m, dx in m, V in mV and time in ms.

The numerical value of sgm in s^-1^m and in S/m is the same as s^-1^m results from dividing S/m by F/m^2^, which are the units of capacitance per unit area.

**Additional Aspects of the Safety Factor Computation**

The existence of small differences in SF computed with our improved version of the SF (SF_m2_) along the circular wavefront derives from the fact that the direction of wavefront propagation slightly differs from the direction of the axial currents except when the direction of the propagation has an inclination of 45º with the Cartesian axis. The slight inclination of the axial currents with the direction of propagation results in the appearance of components of the axial currents that are perpendicular to the direction of the propagation, although their values are very small. In the example shown in Figure 1, the ratio of the perpendicular to the parallel components of the axial currents to the propagation direction is typically 3%. A more accurate computation of the SF in 2D tissues could be defined by decomposing the axial currents into the parallel and perpendicular components to the direction of the propagation. However, this improvement is hampered by the difficulty of obtaining an extremely accurate value of the direction of propagation.

Supplementary Figure S1 shows the activation sequence and the characterization of the source-sink relationship in an isotropic tissue stimulated with a 0.5 mm radius circular shaped electrode at the center of the tissue using a version of the SF computation that takes into account the inclination of the axial currents with the direction of propagation (SF_m3_). In Supplementary Figure S1A the direction of propagation is theoretically computed as the unitary position vector from the center of the electrode while in Supplementary Figure S1B the direction of the propagation is defined as the unitary gradient of the activation time. Supplementary Figure S1C shows both SF_m3_ and the SF_m2_ (green lines) distributions as a function of the angular coordinate along the wavefront in the control tissue at four instants, 1 ms, 1.5 ms, 2 ms and 4 ms after the onset of the simulation. Black lines correspond the SF_m3_ computed using the theoretical direction of propagation (SF_m3T_) and red lines correspond to the SF_m3_ computed using the direction of propagation inferred from the activation sequence (SF_m3AT_). This figure shows that the SF_3m_ obtained with the theoretical direction of propagation improves the circular symmetry (Supplementary Figure S1A) and reduces the differences in SF along the circular isochronal lines (black lines in Supplementary Figure S1C) compared to the SF_m2_ formulation (Supplementary Figure 1B and green lines in Supplementary Figure 1C). Unfortunately, the direction of propagation can not be theoretically computed in most cases, so it has to be inferred from the activation sequence. When the direction of propagation is obtained from the activation sequence the accuracy of the SF is reduced (see Supplementary Figure S1B and red lines of Supplementary Figure S1C). Figure S2 shows the horizontal component of the unitary vector of the direction of the propagation in the tissue stimulated with a 0. 5 mm radius circular shaped electrode when it is theoretically computed (Supplementary Figure S2A) and when it is obtained from the activation sequence (Supplementary Figure S2B). In the latter case, the time step for membrane potentials recording was decreased from 0.1 ms to 0.01 ms to yield more accurate activation sequence and direction of propagation. In the case of the theoretical direction of propagation (Supplementary Figure S2A), the value of the horizontal component of the unitary vector in the direction of the propagation exclusively depends on the angle with the horizontal line, whereas in the case of the direction of propagation obtained from the activation sequence (Supplementary Figure S2B), small variations in the horizontal component of the unitary vector of the direction of propagation are found in points with the same theoretical direction. The accuracy of the computation of the direction of propagation could be improved by using smaller spatial discretization but it would dramatically increase the computational requirements. Therefore, SF_m2_ is more appropriate for characterizing the source-sink relationship in 2D tissues.

**Anisotropic Tissue**
